# Supplementary material for: Development of a Novel Quinoline Derivative as a P-Glycoprotein Inhibitor to Reverse Multidrug Resistance in Cancer Cells
Source: Biology (Basel). 2019 Oct 1;8(4):75. doi: 10.3390/biology8040075 (PMC6955663; doi:10.3390/biology8040075)
Supplement: Supplementary file 1 [file biology-08-00075-s001.pdf]

biology

MDPI

Development of a Novel Quinoline Derivative 160a as a P-glycoprotein Inhibitor to Reverse Multi-Drug Resistance in Cancer Cells

Supplementary Material

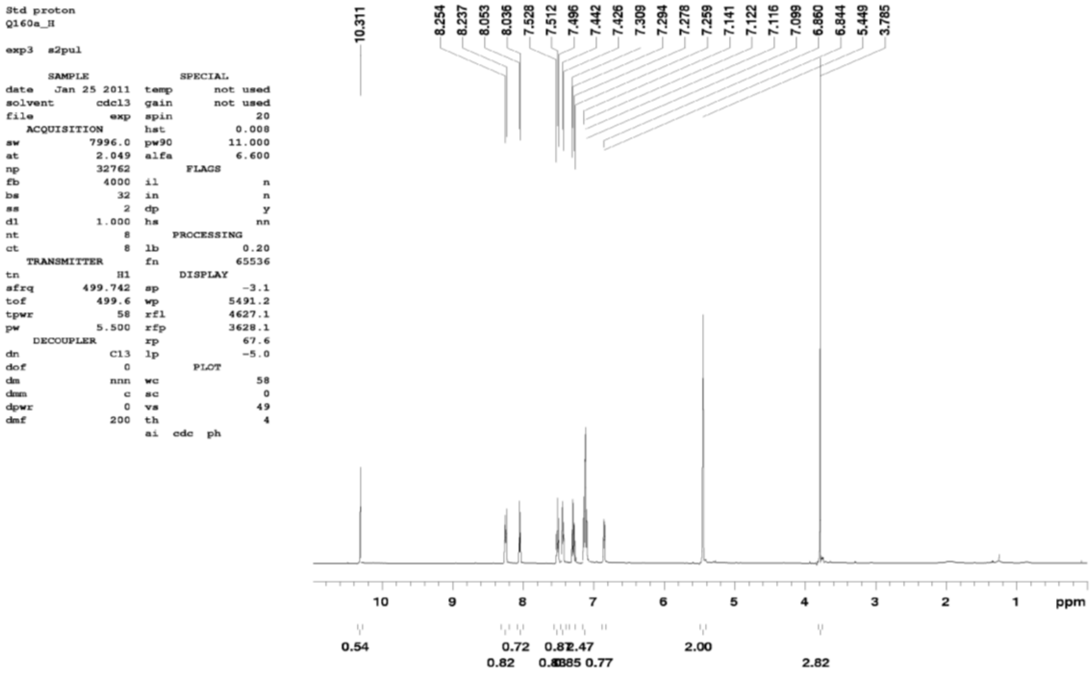

Figure S1. ^{1}H-NMR spectrum of compound 160a. ^{1}H-NMR (500 MHz, $CDCl_3$): δ 3.79 (s, 3H), 5.45 (s, 2H), 6.85 (d, 1H, *J* = 8.0 Hz), 7.12 (t, 3H, *J* = 8.5 Hz), 7.29 (t, 1H, *J* = 7.5 Hz), 7.43 (d, 1H, *J* = 8.0 Hz), 7.51 (t, 1H, *J* = 8.0 Hz), 8.04 (d, 1H, *J* = 8.5 Hz), 8.25 (d, 1H, *J* = 8.5 Hz), 10.31 (s, 1H); ^{13}C-NMR (100 MHz, $CDCl_3$): δ 55.88, 71.63, 111.75, 113.19, 114.12, 118.45, 119.83, 120.60, 130.27, 130.38, 132.04, 137.86, 138.86, 140.95, 152.21, 155.80, 160.61, 194.51; HRMS (ESI): Calcd. for $C_{18}H_{16}NO_3$ $[M+H]^+$, 294.1130; found 294.1118; melting point = 85.6–87.0 °C.

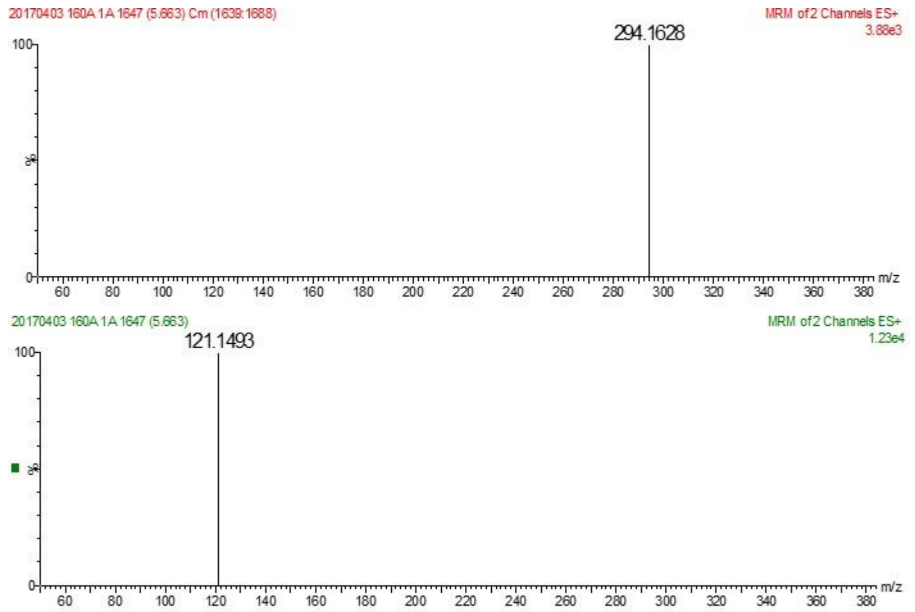

Figure S2. Chromatograph of compound 160a analyzed by UPLC/MS. Parent/daughter mass-to-charge ratio (m/z) is 294.1628/121.1493. The ESI (Electrospray ionization) source was used at positive ion mode. The conditions of MS analysis were designed as follows: capillary voltage: 3.0 kV; source temperature: 150 °C; desolvation temperature: 350 °C; cone gas flow: 45L/H; desolvation gas flow: 800L/H; the cone voltage (cv) and collision energy (CE) were set to match the MRM of each marker. The Masslynx V4.1 software (Waters) has been used for instrument control, data acquisition and handling.

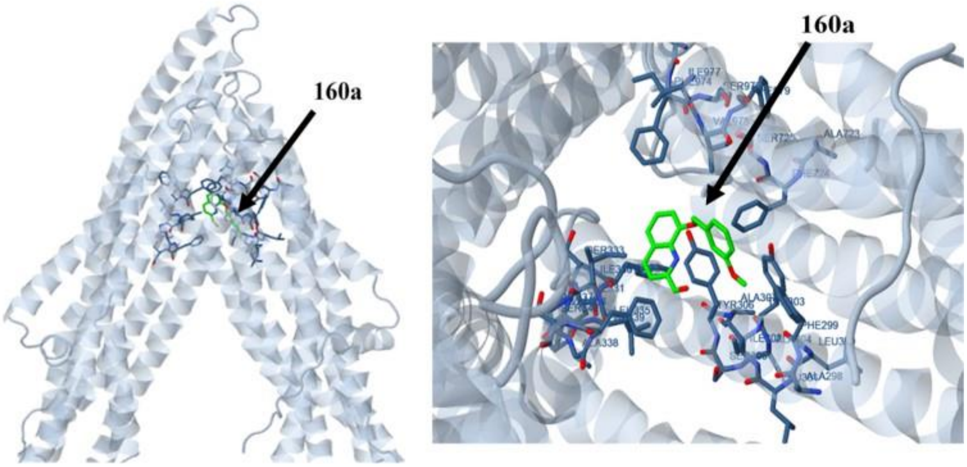

Figure S3. The three-dimensional model of compound 160a interacting with P-glycoprotein obtained in Molecular DockingServer.
